# Fusing Range Measurements from Ultrasonic Beacons and a Laser Range Finder for Localization of a Mobile Robot

**DOI:** 10.3390/s150511050

**Published:** 2015-05-11

**Authors:** Nak Yong Ko, Tae-Yong Kuc

**Affiliations:** 1 Department of Electronics Engineering, Chosun University, 375 Seosuk-dong Dong-gu, Gwangju 501-759, Korea; 2 College of Information and Communication Engineering, Sungkyunkwan University, 300 Cheoncheon-dong Jangan-gu Suwon, Gyeonggi-do 440-746, Korea; E-Mail: tykuc@skku.edu

**Keywords:** fusing measurements, mobile robot, laser range finder, ultrasonic beacons, data association, unscented Kalman filter

## Abstract

This paper proposes a method for mobile robot localization in a partially unknown indoor environment. The method fuses two types of range measurements: the range from the robot to the beacons measured by ultrasonic sensors and the range from the robot to the walls surrounding the robot measured by a laser range finder (LRF). For the fusion, the unscented Kalman filter (UKF) is utilized. Because finding the Jacobian matrix is not feasible for range measurement using an LRF, UKF has an advantage in this situation over the extended KF. The locations of the beacons and range data from the beacons are available, whereas the correspondence of the range data to the beacon is not given. Therefore, the proposed method also deals with the problem of data association to determine which beacon corresponds to the given range data. The proposed approach is evaluated using different sets of design parameter values and is compared with the method that uses only an LRF or ultrasonic beacons. Comparative analysis shows that even though ultrasonic beacons are sparsely populated, have a large error and have a slow update rate, they improve the localization performance when fused with the LRF measurement. In addition, proper adjustment of the UKF design parameters is crucial for full utilization of the UKF approach for sensor fusion. This study contributes to the derivation of a UKF-based design methodology to fuse two exteroceptive measurements that are complementary to each other in localization.

## 1. Introduction

Localization is one of the major concerns for the navigation of mobile robots, and it is a constant topic of interest in the research in mobile robotics. Although many phenomenal research studies and developments for localization have been conducted, localization in practical use often causes considerable glitches in terms of reliability and robustness. No general methodology is available that can deal with the localization problem in a unified and organized manner, which explains why localization is still extensively researched along with simultaneous localization and mapping (SLAM). Approaches to localization depend on the properties or constraints of robot motion, the work environment and the sensors available for a given problem. The major problem in localization is the uncertainty of both the robot motion and sensor measurement. Therefore, probabilistic solutions, such as Kalman filters (KFs) and particle filters (PFs), constitute the principal approaches to this problem.

The range measured by a laser range finder (LRF) and that by ultrasonic beacons have complementary properties. Although LRFs usually provide accurate and reliable range measurements to walls or objects, they often fail to detect glass and flat shiny objects, such as mirrors and flat metals. Localization methods using LRFs compare the measured range and the range calculated from a given map relevant to range detection. Therefore, the methods are often misled to ambiguity in an environment where similar patterns of floor construction exist. Environments with repeated similar configurations often cause more critical localization errors than uncertainty in the range measurement. This problem can be compensated by performing range measurements using ultrasonic beacons.

In the case of a range measured by ultrasonic beacons, the measurement is less accurate and more prone to errors than the range data measured by an LRF. Nonetheless, these beacon measurements provide location information regardless of the repeated similarity in floor construction, which can resolve the ambiguity problem of the method that uses the LRF only. This paper reports a practical approach that fuses these two complementary measurements for the localization of a mobile robot in an indoor environment.

A probabilistic approach is one of the most promising candidates that could provide comprehensive and real-time solutions to the robot localization problem. The probabilistic approach provides information using probability densities [1]. Sensor measurements are inherently noisy, and environments are, to a certain degree, unpredictable. Thus, the process and the result of perception suffer from uncertainty, and the probabilistic approach has attracted more attention to deal with uncertainty in multisensor data fusion [2] and to provide robust autonomy in complex real-world applications [1]. The probabilistic approach computes a probability distribution over what might be the case in an environment instead of generating a single best guess. Further, the probabilistic approach provides a degree of assurance on the estimated result [1].

KFs and PFs constitute the major probabilistic approaches. Research studies on KFs and PFs and their variants for robot localization have been abundant. KF has been well established since the 1960s and has a well-organized implementation structure [3–5]. Because a pure KF is applicable only to linear models with Gaussian uncertainty in both motion and measurement, most KF variants are developed for application to nonlinear models with non-Gaussian uncertainty [3].

Among the large number of variants, extended KF (EKF) and unscented KF (UKF) are popularly implemented. EKF uses an analytical linearization approach involving derivation of the Jacobian of the motion and measurement model. To remedy the problem of limited applicability due to the derivation of the Jacobian and to accurately approximate nonlinearity up to higher order terms, UKF was developed [6–10]. UKF approximates nonlinear systems using the unscented transform, which employs a set of samples called sigma points. In general, UKF can approximate highly nonlinear systems, requiring comparable or a little more (but not so much) computations as the EKF [4]. The performances of UKF and EKF are noticeably comparable in spite of the superior theoretical properties of the UKF [3,11–15].

PF is one of the major approaches in localization, together with KFs [16–18]. PF uses a large number of samples called particles to represent the probabilistic distribution of the estimation and provides robust estimation results. It can deal with nonlinear motion models and non-Gaussian uncertainty in robot motion and sensor measurement. Moreover, it does not require the derivation of the Jacobian. However, it suffers from an exponential increase in computations, called the curse of dimensionality, as the number of state variables increases. The computational efficiency has been generally known to degrade in the order of EKF, UKF and PF, although the estimation performance improves in the same order [19–21].

KFs and PFs have been used for the localization of robots and vehicles. Zhu *et al.* [22] used UKF for the localization of a tracked underwater vehicle. They used the range from the beacons only and dealt only with the additive noise in robot motion and measurement. Ndjeng *et al.* [12] compared the performance of the localization of the variants of KF. GPS location data were used in the correction process of the EKF, UKF, first-order divided difference (DD1) and second-order divided difference (DD2) methods for the localization of a car. Nemra and Aouf [23] proposed a state-dependent Riccati equation (SDRE) nonlinear filter for the localization of an unmanned aerial vehicle. They used INS and GPS for proprioceptive and exteroceptive sensors, respectively, and compared SDRE with EKF and UKF. Galben [13] reported new motion models, called the spherical velocity and spherical odometry-inertia motion models. Mourllion *et al.* [14] compared the predictive step of EKF, UKF, DD1 and DD2 using simulations. They used a car with INS for the simulation. In particular, several problems of the EKF prediction steps are well explained, together with the problem of using inappropriate coordinates.

Research studies that fused sensor data based on the KF approach have been abundant. The method proposed in this paper is different from these methods in that two distinctive exteroceptive measurements are fused by UKF. Assa and Janabi-Sharifi [24] reported a KF-based method that fuses multiple camera images for robust pose estimation to cope with a sensor defect or failure. In contrast to their method, our approach fuses two different types of range measurements and uses UKF. Wei *et al.* [25] used the unscented information filter, a variation of KF, to fuse camera, LRF and GPS measurements. For the fusion, a camera image was preprocessed to yield odometry information. In contrast to our approach, their method uses an information filter and requires a preprocessing by the iterative closest point (ICP) of the LRF measurements to determine translation and rotation. To use a wireless communication facility for indoor localization, the received signal strength indicator is fused with the inertial measurement unit (IMU) using EKF [26,27]. Because IMU intrinsically detects proprioceptive information, the method uses only one type of exteroceptive measurement, whereas the current work derives a UKF-based method that fuses two complementary exteroceptive measurements. Many of the KF-based methods fuse only one type of exteroceptive measurement with the proprioceptive measurements. Marin *et al.* [28] used camera images indoors or GPS outdoors as exteroceptive information together with IMU as proprioceptive data.

Aside from the filtering methods, adaptive estimation [29] and fuzzy logic-based methods [30] have been proposed. The adaptive estimation method [29] fuses vision, odometry and an attitude heading reference system for use in an indoor environment. This method extracts velocity using vision to apply visual information in the adaptive estimation formulation. The fuzzy logic-based method [30] fuses LRF measurements with sonar array range sensor measurement. Unlike the ultrasonic beacons used in our research, the sonar array range sensor detects the range from a sonar transducer array attached on the robot to objects that block the sonar beam. The sonar array range sensor provides the same information as the LRF using a sonar beam instead of a laser beam. The measurements from LRF and the sonar array are not complementary in the sense that they provide range data to objects. In contrast, our method uses two complementary measurements.

Some research studies use PFs for sensor data fusion [31–33]. Perea *et al.* [31] used PF to fuse odometry, IMU, GPS and LRF for localization. The method incorporates GPS measurement into a usual measurement model, which is based on range measurement. In contrast, the UKF-based fusion approach is proposed in this paper. EKF can also be used to fuse LRF measurement into an encoder, IMU and visual odometry. In this case, LRF measurements need to be transformed to translation and rotation data to fit into the EKF formulation [34]. In contrast to EKF, UKF can deal with the LRF measurements in its own form, as presented in the current paper.

The proposed method asynchronously updates the robot location every time measurements from LRF or ultrasonic beacons are available. Fusing multimodal sensor measurements involves incorporating asynchronous sensor measurements. Odometry sensor measurements generally update faster than exteroceptive sensors, such as LRF, sonars and image sensors [32]. In addition, the update rate varies among the exteroceptive sensors. Synchronous implementation of prediction and update in the KF or PF approach results in loss of measurements from sensors with a faster update rate. Asynchronous implementation of prediction and update can make full use of every measurement [32,33]. In the asynchronous implementation, the prediction process operates every time odometry information is available [32]. The update process works only when exteroceptive measurement is available. Thus, two or more prediction steps may iterate between two consecutive applications during the update process. Another approach to deal with asynchronous measurement is interpolation. Kubelka [34] used three methods for interpolation: incremental position, velocity and trajectory approaches. The methods incorporate a 0.3-Hz measurement information into a 90-Hz EKF frame.

Martinelli [15] used a setting that is very similar to our research. They compared the localization performance of EKF and UKF, which use the range measured by an LRF and the range from artificial landmarks. However, they did not fuse both range data. They only compared the result of using the LRF only with that using the landmark only. In addition, they assumed that the correspondence of the range data with the landmark is completely given. The comparison was only made through simulation. Krejsa and Vechet [21] used only the bearing measurement toward visible beacons for indoor localization. PF, UKF and EKF were used, and the localization performance was compared. They also tested the algorithms using simulations and experiments. Dawood *et al.* [35] modified UKF into interacting multiple model UKF (IMM-UKF) to incorporate a 3D geographical information system and video camera data into a GPS-based localization method. The method was also designed for car navigation in urban areas. In case of spacecraft localization using only the bearing information, Giannitrapani *et al.* [20] compared EKF and UKF. They dealt with more general non-additive noise and compared the accuracy and consistency of EKF and UKF. Stroupe *et al.* [36] proposed distributed sensor fusion for object position estimation using multiple robots. They demonstrated the performance of the method in object location estimation and tracking for robotic soccer.

Most of the research studies listed in the previous paragraph use only one type of exteroceptive measurements. Some of the previous works deal only with the motion model of the location estimation [13,14]. Some of them regard only additive noise [22], whereas non-additive uncertainty is more general in practical systems. Many of them focus on the localization of a car with GPS [12,35]. In the case of indoor localization using beacons, correspondence between the range data and a beacon is provided [15]. Most of the previous studies deal with sensor fusion only in terms of fusion of the proprioceptive and exteroceptive measurements [15] or measurements from multiple agents [36], which do not mean the fusion of measurements from heterogeneous sensors, such as LRF and beacons.

The present study deals with a method of fusing two complementary measurements for indoor localization. The method does not assume correspondence of the range data to a beacon. It uses the UKF approach with non-additive uncertainty in robot motion and measurement. Because one of the measurements comes from the LRF, using EKF in our approach would be inappropriate. The performance improvement by this method is verified through experiments. Whereas many papers compare the variants of KF and PF [3,6,11–15,19–21], the current study focuses on the comparison of the measurement fusion with the results, which use LRF only or the ultrasonic beacon only.

Through experiments, we verify that even though ultrasonic beacons are sparsely populated, have large error and have a slow update rate, they improve the localization performance when fused with the LRF measurement. Similarly, the fusion of LRF measurements significantly improves the performance over the use of ultrasonic beacons only, even though the data association does not improve because of the large uncertainty in the measurements of ultrasonic beacons. Further, proper adjustment of the design parameters of the UKF is crucial for full utilization of the UKF approach for sensor fusion. This paper reports a UKF-based design methodology to fuse two exteroceptive measurements that are complementary to each other in terms of localization.

Section 2 formulates the problem in a manner suitable for sensor fusion through the UKF approach. Section 3 explains the proposed approach from prediction to data association and correction. Section 4 shows the results of the experiments using a mobile robot with an LRF in an indoor environment with ultrasonic beacons. The experiments compare the localization performance of the proposed fusing method with the method that uses LRF only or ultrasonic beacons only. The experiments also show the effect of tuning parameter values on the performance. Finally, Section 5 states the concluding remarks.

## 2. Problem Formulation

### 2.1. Nomenclature

The notations used for the derivation of the fusion method are listed as follows:
**x**(*t*)pose of a robot at time *t*; **x**(*t*) = (*x*(*t*), *y*(*t*), *θ*(*t*))*^T^**θ*(*t*)heading angle of the robot at time *t**N_W_*number of range data measured by an LRF; in this study, *N_W_* = 19
$r_{i}^{W}\left(t\right)$the *i*-th range data from a robot to the walls measured by an LRF**z***^W^*(*t*)vector of the measured range data by an LRF; 
$z^{W}\left(t\right)={\left(r_{1}^{W}\left(t\right),r_{2}^{W}\left(t\right),\cdots ,r_{19}^{W}\left(t\right)\right)}^{T}$
$n_{i}^{W}\left(t\right)$noise of measurement 
$r_{i}^{W}\left(t\right)$**n***^W^*(*t*)noise vector of the measurement by an LRF; 
$n^{W}\left(t\right)={\left(n_{1}^{W}\left(t\right),n_{2}^{W}\left(t\right),\cdots ,n_{19}^{W}\left(t\right)\right)}^{T}$*N_B_*number of ultrasonic beacons; in this study, *N_B_* = 4
$r_{i}^{B}\left(t\right)$range from a robot to an ultrasonic beacon**z***^B^*(*t*)vector of the range data from a robot to the ultrasonic beacons; 
$z^{B}\left(t\right)={\left(r_{1}^{B}\left(t\right),r_{2}^{B}\left(t\right),r_{3}^{B}\left(t\right),r_{4}^{B}\left(t\right)\right)}^{T}$
$n_{i}^{B}\left(t\right)$noise of measurement 
$r_{i}^{B}\left(t\right)$**n***^B^* (*t*)noise vector of the measurements by ultrasonic beacons; 
$n^{B}\left(t\right)={\left(n_{1}^{B}\left(t\right),\cdots ,n_{4}^{B}\left(t\right)\right)}^{T}$*N_ext_*number of all exteroceptive range measurement data; *N_ext_* = *N_W_* + *N_B_* = 23**z**(*t*)exteroceptive measurement data encompassing the range data **z***^W^*(*t*) and **z***^B^*(*t*); 
$z\left(t\right)={\left(z^{B}\left(t\right),z^{W}\left(t\right)\right)}^{T}={\left(r_{1}^{B}\left(t\right),\cdots ,r_{4}^{B}\left(t\right),r_{1}^{W}\left(t\right),\cdots ,r_{19}^{W}\left(t\right)\right)}^{T}={\left(r_{1}\left(t\right),\cdots ,r_{23}\left(t\right)\right)}^{T}$**n**(*t*)vector of exteroceptive measurement noise **n***^W^*(*t*) and **n***^B^*(*t*); 
$n\left(t\right)={\left(n^{B}\left(t\right),n^{W}\left(t\right)\right)}^{T}={\left(n_{1}^{B}\left(t\right),\cdots ,n_{4}^{B}\left(t\right),n_{1}^{W}\left(t\right),\cdots n_{19}^{W}\left(t\right)\right)}^{T}={\left(n_{1}\left(t\right),\cdots n_{23}\left(t\right)\right)}^{T}$**x***^Bi^*location of the *i*-th ultrasonic beacon; **x***^Bi^* = (*x^Bi^*, *y^Bi^*), *i* = 1, 2, 3, 4**x***^B^*location vector of all ultrasonic beacons; **x***^B^* = (**x***^B^*^1^, **x***^B^*^2^, **x***^B^*^3^, **x***^B^*^4^)*^T^**υ*(*t*)linear (translational) velocity of a mobile robot at time *t**ω*(*t*)rotational velocity of a mobile robot at time *t***u**(*t*)robot velocity vector at time *t*; **u**(*t*) = (*υ*(*t*), *ω*(*t*))*n^υ^*(*t*)noise of proprioceptive data *υ*(*t*)*n^ω^*(*t*)noise of proprioceptive data *ω*(*t*)**n^u^**(*t*)noise vector of proprioceptive data **u**(*t*); **n^u^**(*t*) = (*n^υ^*(*t*), *n^ω^*(*t*))*^T^***Q**(*t*)error covariance of exteroceptive measurement noise **n**(*t*)**M**(*t*)error covariance of proprioceptive motion noise **n**^u^(*t*)Σ(*t*)error covariance of state **x**(*t*) at time *t**m*map of the workspace that affects the LRF measurements*g*(·)robot motion model that calculates the next robot pose from the current robot pose using linear and rotational velocities; **x**(*t_i_*) = *g*(**u**(*t*), **x**(*t_i_*_−1_))***h***(·)measurement model that maps the robot pose onto the distance to the beacons and walls; **z**(*t*) = (*h*(**x**(*t*), **x***^B^*, *m*)**Z̅***^B_j_^*(*t*)sigma points of the predicted range to beacon *B_j_* at time *t**z̅^B_j_^*(*t*)weighted mean of sigma points **Z̅***^B_j_^* (*t*)**S***^B_j_^* (*t*)weighted error covariance of the range data to beacon *B_j_***K**(*t*)Kalman gain with which the difference between the predicted and actual measurements is multiplied to adjust the predicted pose into best estimationΣ*^x^*^,^*^z^*(*t*)cross-error covariance between the state and measurement**S**(*t*)error covariance of the measurement

Using these notations, we describe the problem and derive the UKF approach to the problem.

### 2.2. Problem Formulation

This study deals with the following localization problem. The state to be estimated is the pose of robot **x**(*t*) at time *t*. Two types of exteroceptive measurements are available: range data to the walls measured by an LRF and range data to the beacons measured by ultrasonic sensors. The LRF on the robot detects the range from the robot to the walls in the environment. There are 19(*N_W_*) range data **z***^W^*(*t*) in which adjacent measurements $r_{i}^{W}\left(t\right)$ and $r_{i+1}^{W}\left(t\right)$ are separated by 10° in the same horizontal scanning plane. The floor map for the workspace is given. There are 4(*N_B_*) ultrasonic beacons, which are called ultrasonic satellites (USATs), that emit an ultrasonic signal. The signal is detected by a receiver on the robot, and the receiver calculates distance **z***^B^*(*t*) to the beacons using the time delay of the signal reception from the signal emission. For notational convenience, we encompass all 23(*N_ext_* = *N_W_* + *N_B_*) exteroceptive measurements as measurement vector **z**(*t*).

The locations of four beacons **x***^Bi^*, *i* = 1,2,3,4 are known, but the information on the correspondence of the beacon to the range data is not given. Thus, a data association method to find the correspondence between the range data and the beacon is also proposed in Section 3.2. The proprioceptive measurement data **u**(*t*) at time *t* can be measured by odometry sensors, such as encoders or resolvers on the robot wheels.

## 3. Fusing Range Measurements from an LRF and the Beacons

To deal with non-additive noise in both the robot motion and range measurement, the state is augmented to include the noise of the proprioceptive and exteroceptive measurements. The proprioceptive measurement noise vector is denoted as **n**^u^(*t*). The exteroceptive measurement noise vector is denoted as **n**(*t*). Therefore, the augmented state becomes **x***^a^*(*t*).

(1) $$x^{a}\left(t\right)={\left(x\left(t\right),n^{u}\left(t\right),n\left(t\right)\right)}^{T}$$

The augmented state vector has 28 elements, which include three elements in **x**(*t*), two elements in proprioceptive measurement noise **n**^u^(*t*) and 23 elements in exteroceptive measurement noise **n**(*t*). We call the pose of robot **x**(*t*) as the intrinsic state, which is different from augmented state **x***^a^*(*t*).

The procedure that fuses the measurements and estimates the robot location consists of the prediction and correction steps, as listed in Table 1. Prediction_step in Line 1 calculates the *a priori* mean *μ̅*(*t*) and *a priori* error covariance Σ̅(*t*) by projecting the previous state mean *μ*(*t* − 1) and error covariance Σ(*t* − 1) using the current proprioceptive information **u**(*t*). Then, the predicted mean *μ̅*(*t*) and error covariance Σ̅(*t*) are corrected to *μ*(*t*) and Σ(*t*) in Line 2 Correction_step by fusing exteroceptive measurements **z**(*t*). The exteroceptive measurements include both the range of the LRF and USATs. Correction_step also uses map information *m*, which is relevant to the LRF measurement. We can notice that the measurement does not provide information on the correspondence of the ultrasonic range measurement to the USAT.

### 3.1. Prediction of Robot Pose and Error Covariance

Prediction_step starts with the calculation of the sigma point matrix using Equation (2).

(2) $$\chi^{a}\left(t-1\right)=\left[\begin{array}{ccc} \mu^{a}\left(t-1\right) & \mu^{a}\left(t-1\right)+\sqrt{L+\lambda }\sqrt{\Sigma^{a}\left(t-1\right)} & \mu^{a}\left(t-1\right)-\sqrt{L+\lambda }\sqrt{\Sigma^{a}\left(t-1\right)} \end{array}\right]$$

In Equation (2), *μ^a^*(*t* − 1) and Σ*^a^*(*t* − 1) are the mean and error covariance of the augmented state at time *t* − 1. λ = *α*^2^(*L* + *κ*) − *L*, where *α* and *κ* are the scaling parameters that determine how far the sigma points are spread from the mean. $\sqrt{\Sigma^{a}\left(t-1\right)}$ is the Cholesky decomposition of covariance Σ*^a^*(*t* − 1). In Equation (2), the sum of a vector and a matrix is defined as the addition of the vector to each column in the matrix. Mean vector *μ^a^*(*t* − 1) is the vector of the mean of the augmented state at time *t* − 1, as expressed by Equation (3).

(3) $$\mu^{a}\left(t-1\right)=\left[\begin{array}{cccccccccc} \mu_{x}\left(t-1\right) & \mu_{y}\left(t-1\right) & \mu_{\theta }\left(t-1\right) & \mu_{\upsilon }\left(t-1\right) & \mu_{\omega }\left(t-1\right) & \mu_{r1}\left(t-1\right) & \mu_{r2}\left(t-1\right) & \cdots & \mu_{r22}\left(t-1\right) & \mu_{r23}\left(t-1\right) \end{array}\right]$$

The mean of the noise of a proprioceptive or an exteroceptive measurement is assumed to be zero, *i.e.*,

(4) $$\mu_{\upsilon }\left(t-1\right)=\mu_{\omega }\left(t-1\right)=\mu_{r1}\left(t-1\right)=\cdots =\mu_{r23}\left(t-1\right)=0.$$

The error covariance Σ*^a^*(*t* − 1) of the augmented state consists of three components: Σ(*t* − 1), **M**(*t* − 1) and **Q**(*t* − 1).

(5) $$\Sigma^{a}\left(t-1\right)=\left[\begin{array}{ccc} \Sigma \left(t-1\right) & 0 & 0\\ 0 & \text{M}\left(t-1\right) & 0\\ 0 & 0 & \text{Q}\left(t-1\right) \end{array}\right]$$

Σ(*t* − 1) is the error covariance of intrinsic state **x**(*t* − 1) at time *t* − 1. **M**(*t* − 1) is the error covariance of proprioceptive motion noise **n**^u^ (*t* − 1) and describes the motion uncertainty of the robot. It is modeled as Equation (6).

(6) $$\text{M}\left(t-1\right)=\left[\begin{array}{cc} \alpha_{1}\upsilon^{2}\left(t-1\right)+\alpha_{1}\omega^{2}\left(t-1\right) & 0\\ 0 & \alpha_{3}\upsilon^{2}\left(t-1\right)+\alpha_{4}\omega^{2}\left(t-1\right) \end{array}\right]$$

The proprioceptive motion noise is a zero mean with error covariance proportional to the squares of the translational and rotational velocities [37]. Equation (6) shows that the noise of the translational velocity and that of the rotational velocity are not correlated. Parameters *α*_1_, ⋯, *α*_4_ determine the dependence of the error covariance value on the linear and angular velocities. Matrix **Q**(*t* − 1) is the error covariance of exteroceptive measurement noise **n**(*t* − 1).

(7) $$\begin{array}{c} \text{Q}\left(t-1\right)=\left[\begin{array}{cc} {\text{Q}}^{B}\left(t-1\right) & 0\\ 0 & {\text{Q}}^{W}\left(t-1\right) \end{array}\right]\\ {\text{Q}}^{B}\left(t-1\right)=\text{diag}\left[\sigma_{n_{i}^{B}}\right],i=1,2,\cdots ,4\\ {\text{Q}}^{W}\left(t-1\right)=\text{diag}\left[\sigma_{n_{i}^{W}}\right],i=1,2,\cdots ,19 \end{array}$$

In Equation (7) Q*^B^* (*t* − 1) describes the error covariance of **n***^B^*(*t* − 1) of the range measurements from the USATs and Q*^W^* (*t* − 1) denotes the error covariance of **n***^W^* (*t* − 1) of the range data from the LRF. The exteroceptive measurement noise is assumed to be Gaussian with a zero mean, and no correlation exists between the measurements from the USATs and those from the LRF.

In Equation (2), each column of *χ^a^*(*t* − 1) is a sigma point and is decomposed as follows.

(8) $$\chi^{a}\left(t-1\right)={\left[\begin{array}{cccc} \chi^{x}\left(t-1\right) & \chi^{n^{u}}\left(t-1\right) & \chi^{n^{\text{B}}}\left(t-1\right) & \chi^{n^{\text{W}}}\left(t-1\right) \end{array}\right]}^{T}$$

In Equation (8), *χ***^x^**(*t* − 1) corresponds to the three elements of the robot pose, *χ***^n^***^^u^^*(*t* − 1) corresponds to the two elements of the noise of the translational and rotational velocities, *χ***^n^**^^**B**^^ (*t* − 1) corresponds to the noise of the range measurements by the USATs and *χ*^**n**^**w**^^ (*t* − 1) corresponds to the noise of the range measurements by the LRF. Therefore, each sigma point has 28 elements with a dimension *L* = 28 of the augmented state. 2*L* + 1 = 57 sigma points are generated. Therefore, the dimension of sigma point matrix *χ^a^*(*t* − 1) is 28 × 57. λ is the tuning parameter that determines how far away are the sigma points from the mean of sigma point *μ^a^* (*t* − 1) [7,9].

Once the sigma points have been generated by Equation (2), each sigma point is propagated to the state at time *t*, by the robot motion model *g*(·) of Equation (9).

(9) $${\bar{\chi }}^{x}\left(t\right)=g\left(u\left(t\right)+\chi^{n^{u}}\left(t-1\right),\chi^{x}\left(t-1\right)\right)$$

The robot motion model *g*(·) transforms a sigma point to a predicted robot pose using proprioceptive information of the translational and rotational velocities **u**(*t*), which are added by noise element *χ***^n^u^^**(*t* − 1) of the translational and rotational velocities. *g*(·) is expressed by Equations (10) and (11).

(10) $$x\left(t_{i}\right)=g\left(u\left(t\right),x\left(t_{i-1}\right)\right)$$

(11) $$\left(\begin{array}{c} x\left(t_{i}\right)\\ y\left(t_{i}\right)\\ \theta \left(t_{i}\right) \end{array}\right)=\underset{g\left(u\left(t\right),x\left(t_{i-1}\right)\right)}{\underset{︸}{\left(\begin{array}{c} x\left(t_{i-1}\right)\\ y\left(t_{i-1}\right)\\ \theta \left(t_{i-1}\right) \end{array}\right)+\left(\begin{array}{c} -\frac{\upsilon }{\omega }\sin\theta \left(t_{i-1}\right)+\frac{\upsilon }{\omega }\sin\left(\theta \left(t_{i-1}\right)+\omega \cdot \left(t_{i}-t_{i-1}\right)\right)\\ \frac{\upsilon }{\omega }\cos\theta \left(t_{i-1}\right)-\frac{\upsilon }{\omega }\cos\left(\theta \left(t_{i-1}\right)+\omega \cdot \left(t_{i}-t_{i-1}\right)\right)\\ \omega \cdot \left(t_{i}-t_{i-1}\right) \end{array}\right)}}$$

The transformed sigma points are weighted and added to estimate *a priori* mean *μ̄*(*t*) and error covariance Σ̄(*t*).

(12) $$\bar{\mu }\left(t\right)=\sum_{i=0}^{2L}w_{i}^{\left(m\right)}\cdot {\bar{\chi }}_{i}^{x}\left(t\right)$$

(13) $$\bar{\Sigma }\left(t\right)=\sum_{i=0}^{2L}w_{i}^{\left(c\right)}\cdot \left({\bar{\chi }}_{i}^{x}\left(t\right)-\bar{\mu }\left(t\right)\right){\left({\bar{\chi }}_{i}^{x}\left(t\right)-\bar{\mu }\left(t\right)\right)}^{T}$$

Here, ${\bar{\chi }}_{i}^{x}\left(t\right)$ represents the *i*-th column of matrix *χ̄***^x^**(*t*). $w_{i}^{\left(m\right)}$ and $w_{i}^{\left(c\right)}$, *i* = 0, 1, ⋯, 2*L* are the weights of the calculations of the mean and covariance using the sigma points, which can be set using the unscented transformation (UT) or scaled UT (SUT) formulation [9,10]. In our implementation, SUT is used and is expressed as Equation (14).

(14) $$\begin{array}{c} w_{0}^{\left(m\right)}=\frac{\lambda }{L+\lambda }\\ w_{0}^{\left(c\right)}=\frac{\lambda }{L+\lambda }+\left(1-\alpha^{2}+\beta \right)\\ w_{0}^{\left(m\right)}=w_{0}^{\left(c\right)}\frac{1}{2\left(L+\lambda \right)},i=1,\cdots ,2L\\ \lambda =\alpha^{2}\left(L+\kappa \right)-L \end{array}$$

Once the weights in Equation (14) are determined, they should be kept and used throughout the remaining UKF procedure. Mean *μ̄*(*t*) and covariance Σ̄(*t*) are adjusted in Correction_step using the measurement to yield a posterior (after the measurement update) state and covariance estimates.

### 3.2. Association of a Beacon Range Measurement to a Beacon

To utilize the range data from a robot to the beacons, associating each range data to a corresponding USAT beacon is necessary. Table 2 lists the data association procedure. Lines 1–5 calculate the predicted mean and error covariance of the range data to every beacon. Line 2 calculates the range to beacon *B_j_* from the predicted robot pose *χ̄***^x^**(*t*). The measurement function *h_B_j__* (·) maps robot location *χ̄***^x^**(*t*) to the distance from the robot to the beacon at **x***^B_j_^*. Sigma point *χ*^**n***^B_j_^*^ (*t* − 1), which corresponds to the noise of the range measurements to the *j*-th USAT, is added to the range. Then, sigma points **Z̄***^B_j_^* (*t*) of the predicted range are calculated, as shown in Line 2. Line 3 calculates mean *z̄^B_j_^* (*t*) of the predicted range, and Line 4 calculates error covariance **S***^B_j_^*(*t*) of the range data to beacon *B_j_*. Lines 6–11 find the corresponding beacon to every range measurement that maximizes the probability that the measured range is near the predicted range data calculated in Lines 1–5. For every measurement $r_{k}^{B}\left(t\right)$, (k=1,2,3,4), a correspondence *c_k_*(*t*), (*k* = 1, 2, 3, 4), is assigned.

### 3.3. Correction of the Predicted Pose and Error Covariance

The correction procedure is otherwise called the measurement update, because it corrects predicted pose *μ̄*(*t*) and covariance Σ̄(*t*) using the exteroceptive measurements. It adjusts the predicted pose using the weighted difference between the actual and the predicted measurement values. Weight **K**(*t*), called the Kalman gain, is calculated by Equation (15).

(15) $$\text{K}\left(t\right)=\Sigma^{x,z}\left(t\right)S\left(t\right)$$

Σ*^x^*^,^*^z^*(*t*) is the cross-error covariance between predicted state *χ̄***^x^**(*t*) of Equation (9) and the predicted measurement. **S**(*t*) is the error covariance of the predicted measurement. The predicted measurement is calculated using measurement function *h*(·).

(16) $$\bar{Z}\left(t\right)={\left(h\left({\bar{\chi }}^{x}\left(t\right),x^{B},m\right)+\chi^{n}\left(t-1\right)\right)}_{\left(23\times 57\right)}$$

Measurement function *h*(·) maps robot pose *χ̄***^x^**(*t*) onto the distance to the beacons and walls considering beacon location **x***^B^* and floor map *m*. *h*(·) is a function that calculates the distance from the robot to the walls in the LRF measurement case. In the USAT measurement case, it calculates the distance from the robot to the beacons. Thus, the function requires the robot location, locations of the beacons and floor map *m* as its argument. Map *m* takes on the information related to the LRF measurement. In the implementation, map *m* is represented by grids. A grid has one of two attributes: occupied or free. A grid is occupied if some object that blocks the laser beam from the LRF occupies the grid.

In Equation (16), exteroceptive noise *χ***^n^**(*t* − 1) includes the measurement noise of both the USAT and LRF.

(17) $$\chi^{n}\left(t-1\right)={\left(\chi^{n^{B}}\left(t-1\right),\chi^{n^{W}}\left(t-1\right)\right)}^{T}$$

Equation (16) indicates that the predicted measurement is the addition of the measurement calculated by measurement function *h*(·) and exteroceptive measurement noise *χ***^n^**(*t* − 1). Measurement **Z̄**(*t*) comprises all 23 range measurements by the USATs and LRF. Mean **z̄**(*t*) of the predicted measurements is the weighted sum of the predicted measurements.

(18) $$\bar{Z}\left(t\right)={\left(\sum_{i=0}^{2N}w_{i}^{\left(m\right)}{\bar{Z}}_{i}\left(t\right)\right)}_{\left(23\times 1\right)}$$

Error covariance **S**(*t*) and cross-error covariance Σ*^x^*^,^*^z^* (*t*) are calculated as follows.

(19) $$\text{S}\left(t\right)={\left(\sum_{i=0}^{2N}w_{i}^{\left(c\right)}\left({\bar{Z}}_{i}\left(t\right)-\bar{z}\left(t\right)\right){\left({\bar{Z}}_{i}\left(t\right)-\bar{z}\left(t\right)\right)}^{T}\right)}_{\left(23\times 23\right)}$$

(20) $$\Sigma^{x,z}\left(t\right)={\left(\sum_{i=0}^{2N}w_{i}^{\left(c\right)}\left({\bar{\chi }}_{i}^{x}\left(t\right)-\bar{\mu }\left(t\right)\right){\left({\bar{Z}}_{i}\left(t\right)-\bar{z}\left(t\right)\right)}^{T}\right)}_{\left(3\times 23\right)}$$

Using the Kalman gain (15), predicted location mean *μ̄*(*t*) and covariance Σ̄(*t*) are adjusted to the final estimated values *μ*(*t*) and Σ(*t*).

(21) $$\mu \left(t\right)=\bar{\mu }\left(t\right)+K\left(t\right)\left(z\left(t\right)-\bar{z}\left(t\right)\right)$$

(22) $$\Sigma \left(t\right)=\bar{\Sigma }\left(t\right)-K\left(t\right)S\left(t\right)K^{T}\left(t\right)$$

This process ends the estimation of robot location *μ*(*t*) and its error covariance Σ(*t*).

### 3.4. Application of the Method

This section explains the issues of the application of the method. The number of elements in the augmented state in Equation (1) is 28. This number includes the following elements: three elements for the robot pose, two elements for the robot velocity, four (*N_B_* = 4) USAT measurements and 19(*N_W_* = 4) LRF measurements. According to Equations (2) and (8), 57 sigma points are generated. The distribution of the sigma points represents the probabilistic distribution of the augmented state as the particles in PF represent the probabilistic distribution of the state. The sigma points are deterministically sampled, whereas the particles are sampled in a random manner provided that the sampling satisfies the motion model and motion uncertainty properties. Usually, the number of particles amounts to several hundreds to thousands. A small number of sigma points compared with those of the particles contribute to the efficient computation time of the UKF approach.

The performance of the UKF method is partly affected by the selection of SUT parameters *α*, *κ* and *β* in Equations (2) and (14). In particular, in the indoor localization case, these values should be properly selected to guarantee that the sigma points are located within the same compartment where the robot is located. Measurement function *h*(·) for the sigma points at different compartments uses local map information, which is different from that viewed by the robot. This difference causes incorrect measurement prediction in the application of Equation (16), which happens when a too large *α* value is used. In the implementation, which is explained in Section 4, the following parameter values are used.

(23) $$\begin{array}{ccc} \alpha =0.6, & \kappa =0, & \beta =2.0 \end{array}$$

USAT uses ultrasound signals with a frequency of 40 kHz, and its measurement error covariance, shown in Equation (7), is $\sigma_{n_{i}^{B}}$, whereas that of the LRF is $\sigma_{n_{i}^{W}}$. Moreover, the USAT measurement is updated at every 625 ms, whereas the LRF measurement is updated at every 22 ms. In addition, only four ultrasonic beacons are sparsely populated in the work space. Therefore, the USAT measurements might appear to deteriorate the localization performance if used with the LRF measurement. On the contrary, as will be shown in Section 4, the USAT measurements still improve the localization performance when fused with the LRF measurement.

## 4. Experiment and Performance Evaluation

### 4.1. Experiment Setup and Implementation

**Figure 1:**
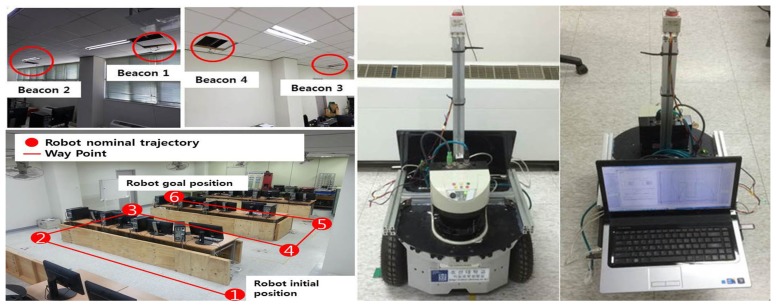
Set up of the experiment: robot, sensors and work area.

**Figure 2:**
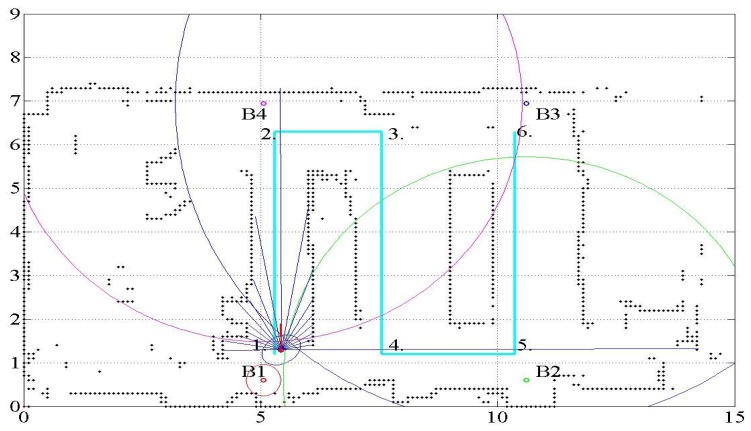
Work area and trajectory of the experiment. B, beacon.

The proposed method is verified by an experiment using a differential drive robot in an indoor environment. The methods are tested using a dataset that is generated by the experiment. Figure 1 shows the setup of the navigation environment, sensors and robot. The work area is a classroom with a dimension of 15 m × 8 m. Tables are present in the work area where desktop computers are located. Partitions are used to make corridors in the classroom. A grid map is independently generated from the experiment. It is generated by a modified ICP method with some manual correction. The robot navigates through waypoints connected in straight lines from Locations 1–6, as shown in Figure 2. The differential drive robot used in the experiment is “RP-NRLAB02” of Redone Technologies, Korea. The proprioceptive information of translational velocity *υ* and rotational velocity *ω* is calculated from the rotation angle sensed by the resolvers on the wheel drive motors. In the experiment, the robot is manually controlled using a joystick to follow the nominal path. A map and a nominal path are made and marked on the floor for manual tracking. The robot is manually placed at its initial location.

The ultrasonic beacon system comes from the Korean company, LPS, and is called “USAT A105”. It consists of four transmitters at the ceiling and one receiver in the robot. The transmitters are indicated as “Beacon” in Figure 1, and the receiver is located at the top of the post on the deck of the robot. The USAT 105 uses a 40-kHz ultrasonic signal. The maximum detection range is 15 m in an open-air environment without any obstruction between the beacon and the receiver. The measurement rate is 1.6 Hz. Although the range error bound listed in the specification table is 10 mm, the standard deviation of the range measurement error is reported to be around 40 cm according to the test result in our laboratory. A 315-MHz RF signal from an amplitude-shift keying (ASK) transmitter is used for synchronization. After all ultrasound signals from the four beacons are received, all distance data are calculated and used for localization.

The USAT 105 provides the correspondence between the measurement and the beacon. However, the correspondence data are not used in the implementation, because this research is designed for more general problems, where correspondence is not given. In some other ultrasonic range measurement systems, the signals from the beacons are not individually coded, and the correspondence is not given.

The LRF on the deck of the robot is “LMS-511”, which is a product of the SICK company. The LRF uses an invisible infrared light with a wavelength of 905 nm and can detect at a maximum distance of 80 m at an update rate of 25–100 Hz, depending on the mode setting. The angular scanning range extends to 190° with a minimum scanning angular resolution of 0.25°. The measurement error shown in the specification is within 50 mm.

The algorithm runs on a computer with CPU Intel(R) Core(TM) i7-3770 at a speed of 3.40 GHz. The computer has 8.00 GB of memory and works on a 32-bit Microsoft Windows 7 Enterprise K operating system. The algorithm is implemented using MATLAB Version R2014a(8.3). The runtime of an iteration of the algorithm is 8 ms.

The proposed method is implemented in a manner similar to the “asynchronous MCL” method [32]. In our implementation, if both the LRF and USAT measurements are available in an algorithm runtime period, they are simultaneously processed as described in this paper. If only one measurement is available, then only the available sensor is used for the update step, whereas the other missing measurement is ignored in the framework presented in this paper. In other words, the formulation related to the measurement that is missing is deleted in the process. For example, if USAT measurements are missing and only the LRF measurements are available in an instant, then measurement **z**(*t*) consists of only **z***^W^*(*t*) and excludes **z***^B^*(*t*). Measurement noise **n**(*t*) includes only **n***^W^*(*t*) and not **n***^B^*(*t*). Likewise, all other formulations discard the terms related to the USAT measurements. In our implementation, the update of the LRF measurement runs more frequently than that of the USAT measurement. Approximately, the update of LRF runs every 22 ms, whereas that of the USAT runs at every 620 ms.

Figure 2 shows the floor map, location of waypoints and ultrasonic beacons, together with the robot pose. The black connected dots that display the floor map represent the occupancy grid with a grid size of 10 cm × 10 cm. The dimensions are indicated in meters. The robot is controlled by a joystick to move through the path from Location 1 to Location 6. The initial pose of the robot motion, indicated as Number 1, has the coordinate **x**(*t_s_*) = (5.3 m, 1.21 m, *π*rad). The final pose of the robot motion is **x**(*t_g_*) = (10.26 m, 6.16 m, *π*rad). The *x* and *y* coordinates correspond to the horizontal and vertical directions, respectively. The heading angle represents the angle from the *x*-coordinate axis in the counterclockwise direction. The ultrasonic beacons are attached at the ceiling of the room. As presented in Section 2.2, only the range data from the ultrasonic beacons are given, whereas no information on the beacon identification number associated with the range data is given. In this respect, the method can be said to work under a partially unknown environment.

In Figure 2, the light-blue line shows the nominal path of the robot. The performance of the method is analyzed by the deviation of the estimated robot location from the nominal path. The four small colored (red, green, blue and pink) circles denoted by B1, B2, B3 and B4 represent the ultrasonic beacons. The numbers shown on the nominal path indicate the waypoints. The arcs centered at the beacons indicate the range from the corresponding beacons. The red circle with a line segment represents the estimated robot location. The red line attached to the robot location displays the estimated robot heading. The range detected by the LRF is indicated by the blue lines emitting from the estimated robot location.

### 4.2. Performance Evaluation

To evaluate the performance of the proposed approach of fusing the range data by the UKF approach, two measures are used: distance error of the estimated location from the nominal path and the rate of correct association of the correspondence to USATs. The performance is evaluated using different sets of UKF tuning parameters. In addition, to verify the effect of fusing two types of range measurements, the result is compared with that using only the LRF or USATs.

The experiments use one dataset of sensor measurements. Each method is executed only once. Because UKF distributes the sigma points in a deterministic manner, it provides the same result every time it is applied to the same dataset. The same dataset is used for the execution of the fusion, LRF only and USAT only approaches; thus, the methods are compared under fair conditions.

#### 4.2.1. Effect of Tuning Parameter Values

**Figure 3:**
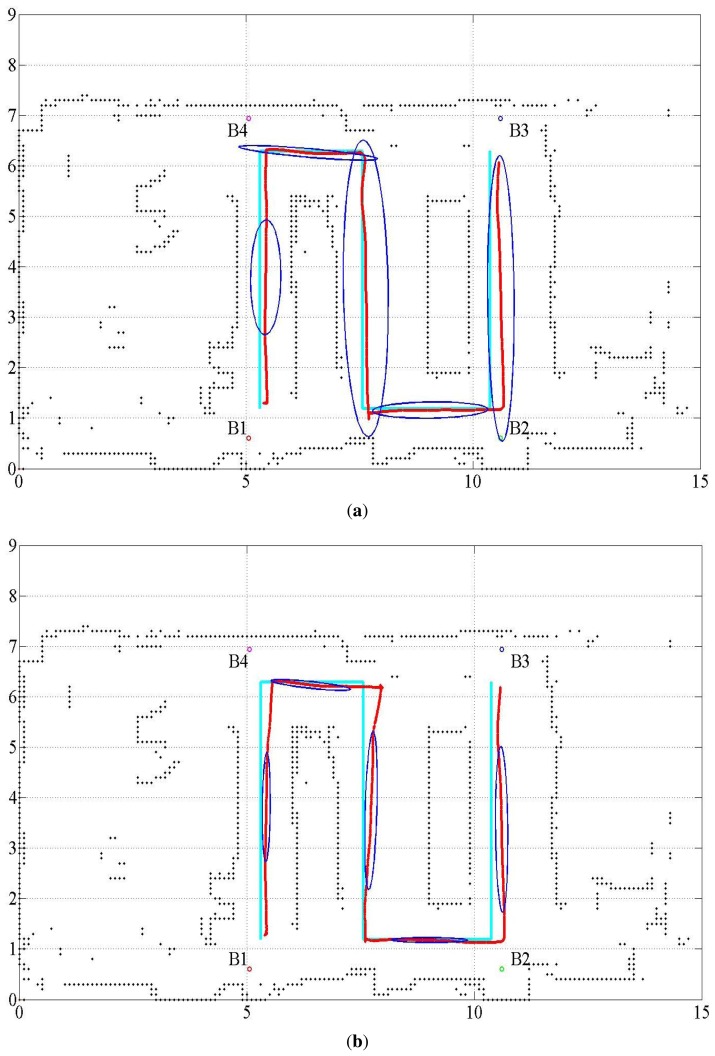

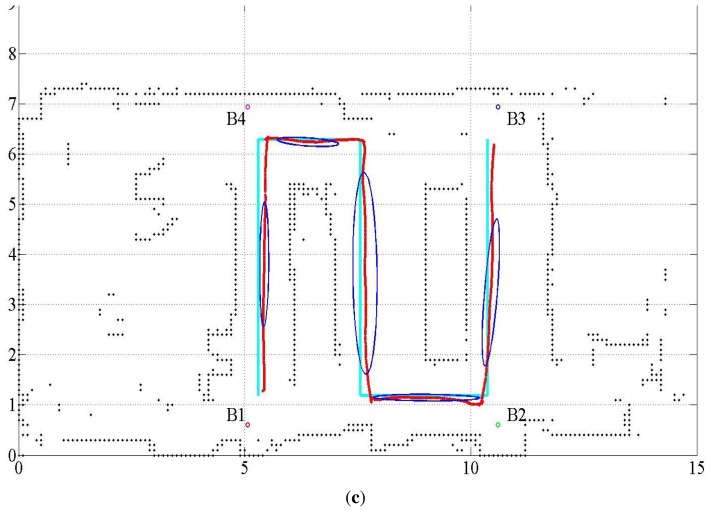
Estimated robot trajectory for the three cases. (**a**) Estimated trajectory for Case 1; (**b**) estimated trajectory for Case 2; (**c**) estimated trajectory for Case 3.

**Figure 4:**
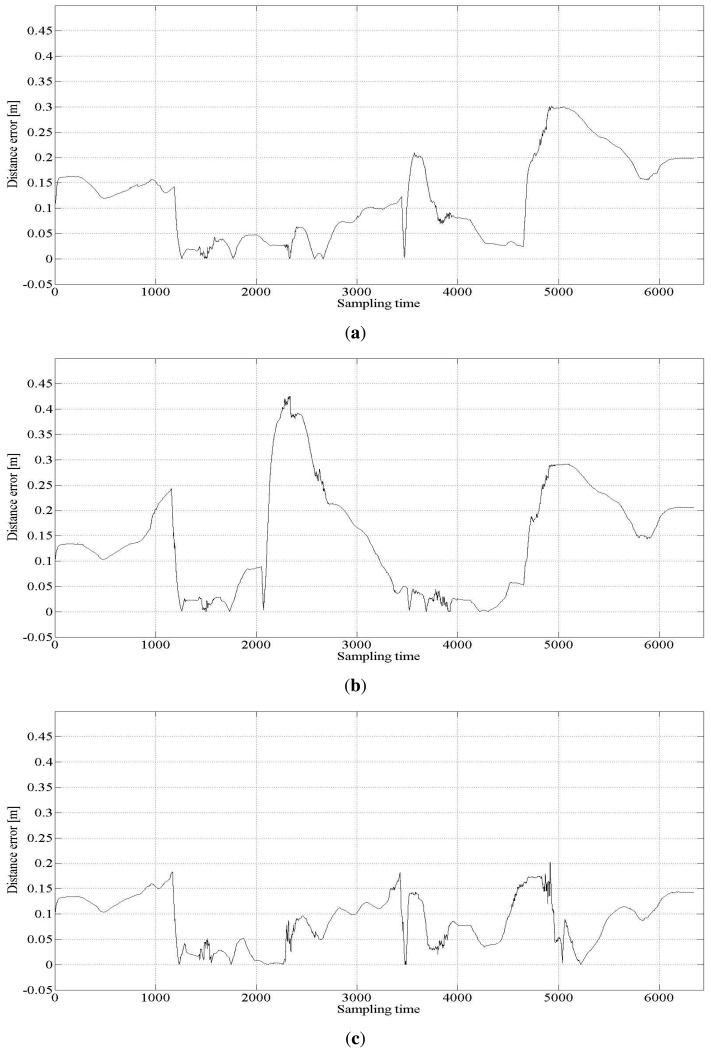
Distance error for the three cases. (**a**) Distance error for Case 1; (**b**) distance error for Case 2; (**c**) distance error for Case 3.

Several tuning parameters are available for the implementation of the UKF. They are α*_i_*, *i* = 1, ⋯, 4 for error covariance M(*t*) of the proprioceptive measurement noise **n^u^**(*t*) and error covariance Q(*t*) of the exteroceptive measurement noise **n**(*t*). Table 3 lists three different sets of tuning parameter values used to investigate the behavior of the result. Figure 3 shows the estimated trajectories for these cases, and Figure 4 shows the distance error of the estimated trajectories. In Figure 3, five error covariance ellipses are observed at the locations indicated by the blue dots at the center of the ellipses. To make the ellipses more clearly distinguishable, they are magnified 300 times. Table 4 lists the statistical evaluation of the distance error of the location estimation. It shows the mean, standard deviation, root mean square and maximum distance error for the three cases. Table 5 lists the rate of successful association of the USAT range data to the beacons.

Table 4 shows that Case 3 outperforms Cases 1 and 2. From the results of Cases 1 and 3, the measurement noise of the USAT should be set larger than that of the LRF measurement. For the best result, the standard deviation of the USAT range measurement noise is set as $\sigma_{n_{i}^{B}}=0.4\text{m}$, whereas that for the LRF is set as $\sigma_{n_{i}^{B}}=0.1\text{m}$. Lowering the standard deviation of the USAT range measurement noise to $\sigma_{n_{i}^{B}}=0.31\text{m}$ degrades the localization performance, as shown in Case 1. Further, from the results of Cases 2 and 3, we conclude that some uncertainty is present in the proprioceptive information of the translational and rotational velocities of the robot. If a lower uncertainty in the proprioceptive measurement is assumed, the localization performance becomes worse, as shown in Case 2.

Only one specific parameter set exists that characterizes the sensor systems. It is heuristically determined through many trial and error processes. The parameter set for Case 3 is the one determined in the experiment. The other two parameter sets that correspond to Cases 1 and 2 are used for the purpose of comparison.

From Table 5, we can also notice that a larger location estimation error also reduces the success rate of the association of the USAT range data to the beacon, because a larger estimation error in the robot location causes a larger discrepancy between the expected distance and the measured actual distance to the beacon.

#### 4.2.2. Comparison of the Sensor Fusion and with No Sensor Fusion

**Figure 5:**
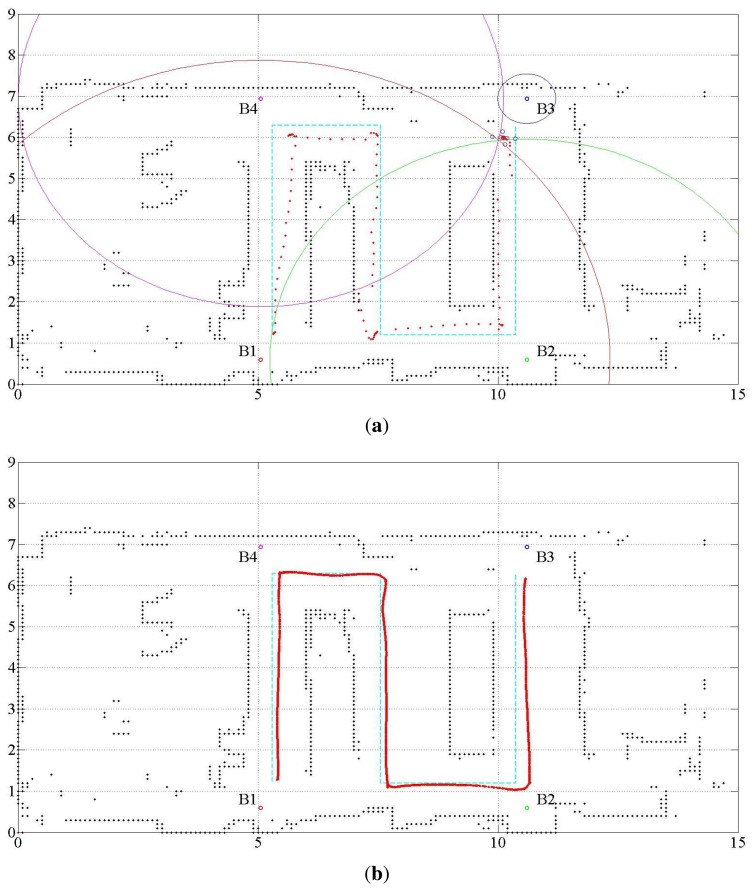
Estimated robot trajectory for the cases with no sensor fusion. (**a**) Estimated trajectory for the case of USAT only (Case 4); (**b**) estimated trajectory for the case of LRF only (Case 5).

**Figure 6:**
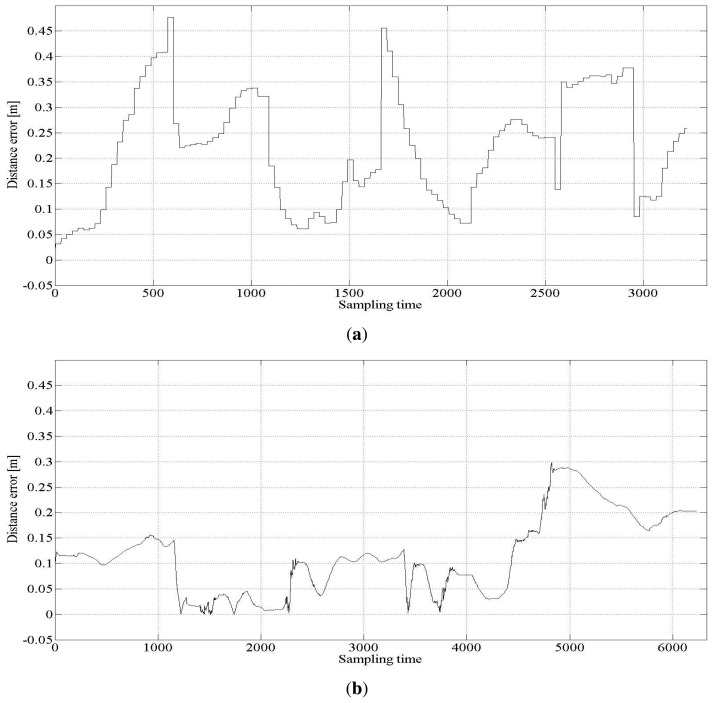
Distance error for the three cases. (**a**) Distance error for the case of USAT only (Case 4); (**b**) distance error for the case of LRF only (Case 5).

The proposed fusion approach is compared with the methods without sensor fusion, which used USATs or LRF only. The statistical evaluation of the distance error in the cases with no sensor fusion is listed in Table 6, where Case 4 represents the result of the USAT only and Case 5 represents that of the LRF only method. Table 6 also lists the result of Case 3 for ease of comparison. Figure 5 shows the localization estimation, and Figure 6 shows the error distance of the USAT only and LRF only cases. Table 7 lists the rate of correct data association. In the LRF only case, no data association exists, and Table 7 does not show any result of the LRF only case. Discussions on the result listed in Table 7 are presented in Section 4.2.3.

#### 4.2.3. Discussions

The USAT range measurement has approximately four-times larger error than the LRF measurement. Further, USAT measures the range at the rate of 1.6-times per second, whereas the LRF scans the range at the rate of 46 scans per second. These make the USAT only estimation (Case 4) much worse than the estimation by fusion or by the LRF only (Case 5). In addition to the large measurement error and low measurement rate, some other errors are present, attributed to the low performance of the USAT only method, namely obscure correspondence, small number of available data, positional dilution of precision (PDOP) and robot velocity.

Only four USAT range data are available, whereas 19 data are available for the LRF. In addition, if the robot is located around or outside the border of the rectangle formed by the USAT beacons, the PDOP problem deteriorates the localization quality. The localization accuracy differs from place to place and depends on the arrangement of the beacon location. To solve the PDOP problem in the localization based on the USAT measurement, the experiment limits the robot work area within the rectangle where the USAT beacons form the vertices, as shown in Figures 2, 3 and 5.

The velocity of the robot also affects the localization accuracy. The influence of the robot velocity is more critical to the USAT only method due to the low measurement rate. Because the interval between measurements is longer, the prediction error becomes larger for the USAT only method compared with the LRF only or fusion approach. The average linear speed of the robot motion in the experiment is 16.6 m/s.

Nevertheless, the USAT measurement improves the localization performance of the LRF only (Case 5) over that of the fusion (Case 3). This advantage is the major contribution of the proposed sensor fusion method. Fusing the USAT measurements with the LRF measurement is crucial in an environment where a repeated similar indoor structure, such as a long corridor, leads astray the localization scheme that uses the LRF measurement only. For example, suppose that two four-way intersections exist with the same road width. When a robot is in one of the intersections, the robot cannot recognize which intersection it is in if it uses the LRF measurements only. Similarly, a robot cannot distinguish two rooms with the same dimensions as each other if the robot uses the LRF measurements only.

As listed in Tables 5 and 7, approximately 19%–27% of the correspondence calculated by the algorithm is false. Two possibilities account for the occurrence of this false correspondence. The first one is that two or more beacons are located at the same distance from the robot. The second one is that some of the range measurements are erroneous. In the first case, the range difference between two or more range data is small, such that the difference is within the uncertainty range of the sensor measurement performance and false correspondence does not degrade the localization performance.

The method that uses the USAT only results in the best success rate of data association, whereas it results in the worst localization among the cases. Even in Case 1, where the USAT range error is presumed to be less than the actual error, the data association is better than that in Case 3, where the best localization result is achieved. The use of LRF together with the USAT improves the localization, whereas it degrades the data association. This result means that erroneous measurement by the USAT is ignored by fusing the LRF measurement with the USAT measurement; thus, localization improves even though the data association does not improve.

The results indicate that proper adjustment of the tuning parameters is vital to take full advantage of the UKF approach for sensor fusion. The adjustment takes the degree of uncertainty into account for successful implementation of the approach. The uncertainties lie in the robot motion or proprioceptive and exteroceptive measurements, such as the USAT and LRF range measurements. As listed in Table 4, the estimation deteriorates if lower parameter values are used. If lower values are used, the algorithm overly trusts the measurement, even though the actual measurement is not reliable enough.

The KF approach is well known to encompass a huge number of research studies and applications. In addition, every KF is said to differ from one another, and no proprietary KF exists [3]. Similarly, UKF can be used in a large number of variations for implementation. Although they belong to one approach, almost every implementation differs from one another, as well as their performance. The performance depends on how the available measurements are fused, which variables are chosen as states and how the uncertainties in motion and measurements are incorporated into the UKF frame. This paper reports a UKF-based design methodology to fuse the LRF and USAT measurements, which are complementary to each other, for localization. In addition, the effect of tuning parameters on the performance is revealed.

## 5. Conclusions

This paper reports a method that fuses two exteroceptive measurements, which are complementary to each other in terms of localization, and discusses some experimental results. The method fuses measurements by an LRF and USATs using the UKF approach. Because the correspondence of the USAT range data to a beacon is not given, the method also finds a beacon that corresponds to a range measurement. The experiments examine the effects of tuning parameter values on the localization performance. The experiments provide statistical evaluation for the localization error: average, standard deviation, root mean square and maximum error distance from the actual path. In addition, the rate of correct data association for the USAT measurement is given.

From the experiments, we prove that location estimation is improved by the fusion of the LRF and USAT measurements. We also explicitly show that appropriate selection of the tuning parameter values is essential for the full utilization of the approach. We can notice that although ultrasonic beacons are sparsely installed and have large error and a slow update rate, they improve the localization performance when fused with the LRF measurement.

The method can be further developed for applications in a dynamic or unknown environment. In some applications, the location of some of the USAT beacons is not given or USAT beacons are detected in real time. The method can augment the state by incorporating the beacon location in the state, and the USAT location can be estimated because the LRF can provide the robot location by itself.

## Figures and Tables

**Table 1:** Localization procedure.

Localization(*μ*(*t* − 1), Σ(*t* − 1), **u**(*t*), **z**(*t*), *m*)
1.(*μ̄*(*t*), Σ̄(*t*)) = Prediction_step(*μ*(*t* − 1), Σ(*t* − 1), **u**(*t*))
2.(*μ*(*t*), Σ(*t*)) = Correction_step(*μ̄*(*t*), Σ̄(*t*), **z**(*t*), *m*)
3.*return*(*μ*(*t*), Σ(*t*))

**Table 2:** Association of a beacon to a range measurement.

Data association	
1:	for all the beacons *B_j_*, *j* = 1, 2, 3,4, do
2:	**Z̄***^B_j_^*(*t*) = (*h_B_j__*(*χ̄***^x^**(*t*)) + *χ***^n^***^^B_j_^^* (*t* − 1))_(1×57)_
3:	${\bar{z}}^{B_{j}}\left(t\right)=\sum_{i=0}^{2L}w_{i}^{\left(m\right)}\cdot {\bar{Z}}_{i}^{B_{j}}\left(t\right)$
4:	$S^{B_{j}}\left(t\right)=\sum_{i=0}^{2L}w_{i}^{\left(c\right)}\cdot \left({\bar{Z}}_{i}^{B_{j}}\left(t\right)-{\bar{z}}^{B_{j}}\left(t\right))\right){\left({\bar{Z}}_{i}^{B_{j}}\left(t\right)-{\bar{z}}^{B_{j}}\left(t\right)\right)}^{T}$
5:	endfor
6:	for all the range measurements $r_{k}^{B}\left(t\right)$, *k* = 1, 2, 3, 4 do
7:	for all the beacons *B_j_*, *j* = 1, 2, 3, 4
8:	$ML_{k}^{B_{j}}=\det{\left(2\pi S^{B_{j}}\left(t\right)\right)}^{-\frac{1}{2}}\cdot \exp\;\left\{-\frac{1}{2}{\left(r_{k}^{B}\left(t\right)-{\bar{z}}^{B_{j}}\left(t\right)\right)}^{T}{\left[S^{B_{j}}\left(t\right)\right]}^{-1}(r_{k}^{B}\left(t\right)-{\bar{z}}^{B_{j}}\left(t\right)\right\}$
9:	endfor
10:	$c_{k}\left(t\right)={\text{argmax}}_{j}\left(ML_{k}^{B_{j}}\right)$
11:	endfor
12:	return *c_k_*(*t*), *k* = 1, 2, 3, 4.

**Table 3:** Tuning parameters related to measurement noise.

**Proprioceptive Measurement Noise**					**Exteroceptive Measurement Noise**	

**Case**	**α_1_**	**α_2_**	***α*_3_**	**α_4_**	$\sigma_{n_{i}^{B}}$**, *i* = 1, ⋯, 4**	$\sigma_{n_{i}^{W}}$**, *i* = 1, ⋯, 19**
1	0.02	0.02	0.02	0.02	0.31	0.1
2	0.01	0.01	0.01	0.01	0.4	0.1
3	0.02	0.02	0.02	0.02	0.4	0.1

**Table 4:** Mean, standard deviation, root mean square and maximum of distance error (dimension in meters).

**Case**	**Mean**	**Standard Deviation**	**RMSE**	**Maximum Error**
1	0.118	0.082	0.144	0.302
2	0.143	0.105	0.178	0.425
3	0.088	0.050	0.101	0.202

**Table 5:** Rate of associating correct correspondence for each beacon range measurement.

**Case**	**Rate of Successful Association**				**Average Rate of Successful Association**

	**B1**	**B2**	**B3**	**B4**	
1	72.7%	86.4%	73.6%	73.6%	77.3%
2	68.2%	81.8%	68.2%	68.2%	73.2%
3	75.4%	85.4%	75.4%	75.4%	77.0%

**Table 6:** Mean, standard deviation, root mean square and maximum of distance error for Cases 3, 4 and 5 (dimensions in meters). LRF, laser range finder; USAT, ultrasonic satellite.

**Case**	**Mean**	**Standard Deviation**	**RMSE**	**Maximum Error**
3: fusion	0.088	0.050	0.101	0.202
4: USAT only	0.218	0.111	0.245	0.477
5: LRF only	0.115	0.076	0.138	0.299

**Table 7:** Rate of associating correct correspondence for each beacon range measurement for Cases 3 and 4.

**Case**	**Rate of Successful Association**				**Average Rate of Successful Association**

	**B1**	**B2**	**B3**	**B4**	
3: fusion	75.4%	75.4%	75.4%	81.8%	77.0%
4: USAT only	87.3%	86.4%	68.2%	83.6%	81.4%
